# Polo-like kinase 4 (PLK4) as a therapeutic target in breast cancer

**DOI:** 10.1093/carcin/bgaf067

**Published:** 2025-10-14

**Authors:** Armen Parsyan, Harjot Athwal, Vasudeva Bhat, Alison L Allan

**Affiliations:** Department of Anatomy and Cell Biology, Schulich School of Medicine & Dentistry, Western University, London, ON N6A 5C1, Canada; Verspeeten Family Cancer Centre, London Health Sciences Centre and London Health Sciences Centre Research Institute, London, ON N6A 5W9, Canada; Department of Oncology, Schulich School of Medicine & Dentistry, Western University, London, ON N6A 5W9, Canada; Department of Surgery, Schulich School of Medicine & Dentistry, London, ON N6A 4V2, Canada; St Joseph’s Health Care, London, ON N6A 4V2, Canada; Department of Anatomy and Cell Biology, Schulich School of Medicine & Dentistry, Western University, London, ON N6A 5C1, Canada; Department of Anatomy and Cell Biology, Schulich School of Medicine & Dentistry, Western University, London, ON N6A 5C1, Canada; Verspeeten Family Cancer Centre, London Health Sciences Centre and London Health Sciences Centre Research Institute, London, ON N6A 5W9, Canada; Department of Oncology, Schulich School of Medicine & Dentistry, Western University, London, ON N6A 5W9, Canada; Department of Anatomy and Cell Biology, Schulich School of Medicine & Dentistry, Western University, London, ON N6A 5C1, Canada; Verspeeten Family Cancer Centre, London Health Sciences Centre and London Health Sciences Centre Research Institute, London, ON N6A 5W9, Canada; Department of Oncology, Schulich School of Medicine & Dentistry, Western University, London, ON N6A 5W9, Canada

**Keywords:** polo-like kinase 4, PLK4, breast cancer, biomarkers, CFI-400945, centrinone, radioresistance

## Abstract

Polo-like kinase 4 (PLK4) is a key kinase regulating centriole duplication, centrosome maturation, cytokinesis and other cellular processes. Growing evidence suggests a critical role of PLK4 in the development and progression of various cancers. In many cancer types, its upregulation leads to pro-oncogenic phenotypes, while its pharmacologic inhibition leads to anticancer effects. Functionally, PLK4 affects cancer cell proliferation, growth, motility, invasion, migration, epithelial-mesenchymal transition, apoptosis and other critical oncogenic processes. In breast cancer, PLK4 is associated with centrosome amplification, aneuploidy and chromosomal instability, promoting invasive phenotypes and resistance to cancer cell death. PLK4 shows great promise as a prognostic and predictive biomarker in breast cancer. It is commonly found to be overexpressed in primary human breast cancers and is associated with poor oncologic outcomes, clinicopathologic parameters, and high-risk subtypes. Various compounds, such as CFI-400945, centrinone B, and others have been developed to inhibit PLK4 activity. Preclinical studies have shown that PLK4 inhibitors lead to decreased proliferation, growth and migration and increased breast cancer cell death. Moreover, PLK4 inhibition can serve to enhance the effects of other treatments, including radiotherapy. Clinical studies have been initiated with some of these compounds in cancer patients, including those with breast cancer. This manuscript discusses the role of PLK4 as a promising therapeutic target in breast cancer, one of the most common causes of morbidity and mortality in women.

## Graphical Abstract

**Graphical Abstract:**
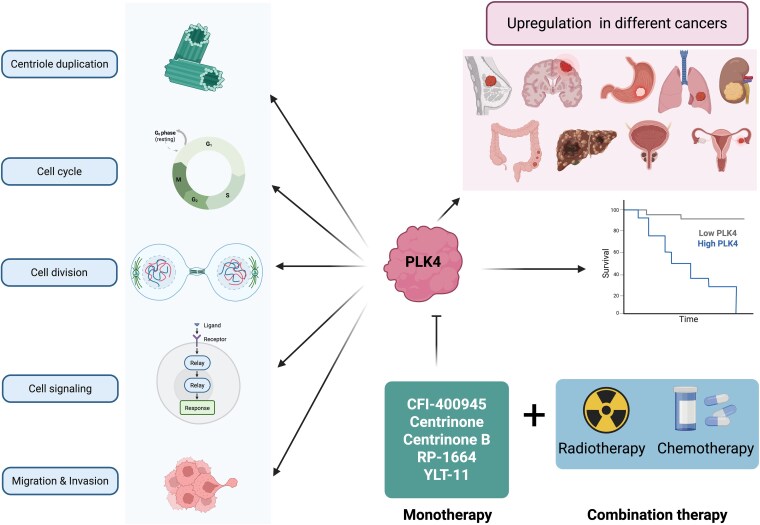

## 1. Introduction

Breast cancer remains one of the most common causes of cancer morbidity and mortality in women [1]. The high degree of tumor heterogeneity and resistance to treatment continue to represent some of the main unmet clinical challenges in breast cancer [2]. Improving breast cancer patients’ treatment and management requires a better understanding of breast cancer biology and the identification of biomarkers and novel targets for drug development [2, 3]. One such emerging molecular target that shows promise as a novel biomarker and drug target is the protein polo-like kinase 4 (PLK4) [4].

**Figure 1.**
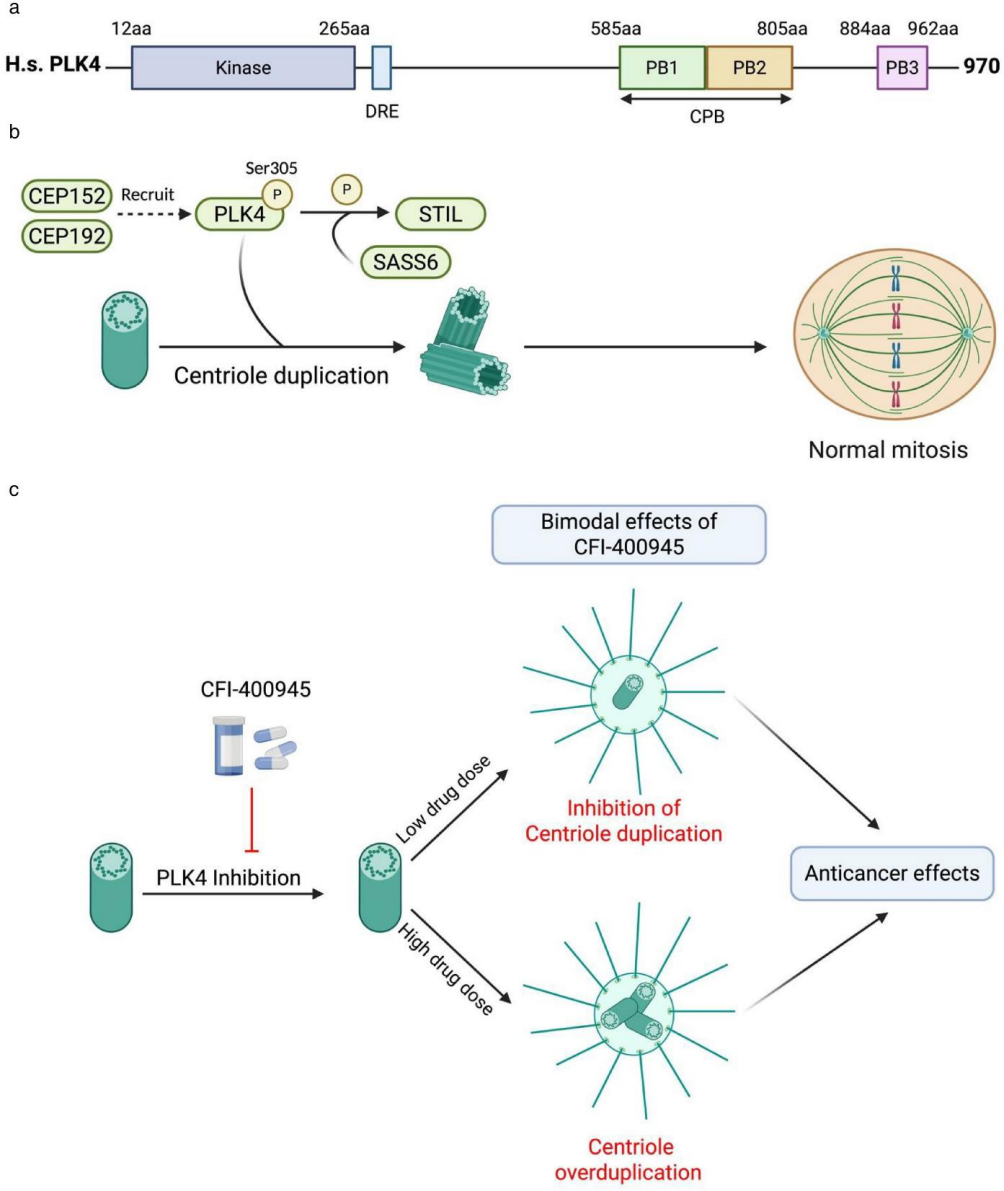
Structure, function, and mechanism of action of the human polo-like kinase 4 (PLK4) protein. (a) The kinase domain is shown in blue (left), and the polo-box domains (PB) in green, orange, and pink (right). When the activity of PLK4 reaches a certain threshold, the protein *trans*-autophosphorylates its downstream regulatory element (DRE), a 24-amino acid region and the E3 ubiquitin ligase recognition site. CPB - cryptic polo box domain; aa - amino acids; H. s.—*Homo sapiens*. Based on [5, 6]. (b) PLK4 is recruited to the centriole by centrosomal protein (CEP) 152 and CEP192 and initiates centriole duplication by interacting with SCL/TAL-interrupting locus protein (STIL) and spindle assembly abnormal protein 6 homolog (SAS-6 or SASS6). Aberrant expression of PLK4 can lead to abnormal centriole duplication and subsequent errors in cell division. (c) The small-molecule inhibitor CFI-400945 inhibits PLK4 by preventing its autophosphorylation at serine 305, but it has a bimodal effect on centriole duplication. At low concentrations, it inhibits centriole duplication and at high concentrations, it causes centriole overduplication. Aberrations in centriole duplication caused by deregulation or inhibition of PLK4 lead to anticancer effects via abnormal cell division and genomic instability (see text). Created with BioRender.com

Human PLK4 is a 970 amino-acid (aa) -long protein that contains an N-terminal kinase domain and C-terminal 3 polo-box domains required for its localization and protein-protein interactions [5, 6] (Fig. 1a). It belongs to a family of PLK serine/threonine kinases, consisting of 5 members (PLK1-5) that play various biological functions, including regulation of centriole duplication, centrosome maturation, cellular division and cell cycle, cytokinesis and others [7]. PLK4 has been identified as a key regulator of centriole duplication [8, 9]. It plays important roles in cell cycle regulation [10], cell division, cell signaling, and motility [11, 12].

Centrioles are microtubule-based organelles comprising the centrosome and play a critical role in nucleating the formation of cilia and flagella [13], organizing microtubules, and building centrosomes that are required for cell division [14]. Centriole duplication is a highly controlled process that prevents the cell from developing multipolar spindles, which can promote chromosomal instability. Detailed mechanisms of PLK4 function and its regulation are reported elsewhere [4, 6]. In brief, centrioles prepare to divide by recruiting PLK4 through centrosomal protein (CEP) 152 [15] and CEP192 [16–18]. PLK4 then binds and phosphorylates STIL (SCL/TAL-interrupting locus protein) [9, 19], which interacts with centrosome-related proteins such as spindle assembly abnormal protein 6 homolog (SAS-6) and centrosomal protein 4.1-associated protein (CPAP) [9, 19] in order to facilitate the process of centriole duplication during the S phase of the cell cycle (Fig. 1b).

Throughout interphase, PLK4 autophosphorylation leads to its degradation, preventing centriole amplification. Degradation of PLK4 is regulated by the SCF/Slimb ubiquitin ligase that recognizes PLK4 after homodimer-dependent *trans*-autophosphorylation of the downstream regulatory element (DRE) [5, 20]. During mitosis, PP2A (Protein Phosphatase 2A^Twins^) counteracts PLK4 autophosphorylation and stabilizes it to promote centriole duplication [21]. Various other complex mechanisms are involved in PLK4 regulation at the epigenetic, transcriptional, translational and post-translational levels [6].

Hereditary mutations of PLK4 lead to phenotypes that might be related to the role of centrioles in biogenesis or cilia, or ciliogenesis [11]. Inherited mutations in PLK4 have been implicated in forms of microcephalic primordial dwarfism such as Seckel syndrome [12], microcephaly, growth failure and chorioretinopathy [11, 22]. PLK4 mutations might be associated with azoospermia in men with Sertoli cell-only syndrome [23].

### 1.1. PLK4 in tumorigenesis and human cancers

Overall, numerous studies of PLK4 support its function as a pro-oncogene (Table 1). As a key regulator of centriole duplication, PLK4 dysregulation leads to various abnormalities in centrosome biogenesis and maintenance [8, 66, 67]. PLK4 downregulation leads to loss of centrioles (the core structures of centrosomes) and prevents their duplication, giving rise to mitotic abnormalities [67]. Acentriolar chromosomes have been suggested to be characteristic of many breast cancers and are more common in its aggressive, TNBC subtype, correlating with advanced stage and grade of the disease [68]. It is unclear whether this observation is a reflection of an intrinsic tumorigenic program or a response of aggressive cancer cells to downregulate their proliferation, since the loss of centrosomes may halt cell proliferation by triggering cell cycle arrest [69]. Upregulation of PLK4 leads to centrosome amplification and extra centrosomes, promoting chromosome mis-segregation and leading to aneuploidy and chromosomal instability, a hallmark of cancer [66]. Indeed, PLK4 plays an important role in the initiation and progression of tumors. In mouse transgenic models with a tumor suppressor p53-null background, overexpression of Plk4 induces centrosome amplification and tissue hyperplasia, leading to continuous cell division and setting up a pro-tumorigenic state [70]. Transient Plk4 overexpression in transgenic mice with a p53-deficient background has also been shown to accelerate tumorigenesis in p53-deficient epidermis associated with centrosome amplification, aneuploidy and multipolar divisions [71]. Furthermore, centrosome amplification has been reported to be sufficient to promote spontaneous tumorigenesis (including lymphoma, squamous cell carcinoma and sarcoma) in mice without transgenic p53 manipulations [72]. In a doxycycline-inducible Plk4 mouse model, chronic or transient increase in Plk4 promotes aneuploidy, mitotic errors, complex karyotypes and centrosome amplification that drives the development of spontaneous tumors in various tissues [72].

**Table 1.** Roles of PLK4 in various nonbreast cancer malignancies and its potential as a biomarker.

Tumor type	Roles in cancer biology, treatment response and as a biomarker	References
Lung cancer	*PLK4* is overexpressed in lung carcinoma and is associated with unfavorable survival; high PLK4 expression correlates with greater tumor size, lymph node metastasis and confers poor survival in non-small cell lung cancer.	[24–26]
Gastric cancer	PLK4 mRNA and protein are commonly found upregulated in gastric cancer; induced overexpression of PLK4 in gastric cancer cells leads to centrosome amplification and chromosomal instability and suppresses primary cilia formation; higher PLK4 expression positively correlates with an advanced stage and, in multivariate analysis, with a shorter disease-free survival (DFS).	[27, 28]
Colorectal cancer	PLK4 protein expression is upregulated in colorectal cancer tissues compared with normal tissues and in colon cancer cell lines compared with a normal epithelial cell line; knockdown of PLK4 results in significant decrease in cell viability, proliferation, xenograft tumor growth in mice, and inactivates the Wnt/β-catenin pathway; through the regulation of the latter, PLK may mediate epithelial-mesenchymal transition (EMT) and cancer cell invasive and migratory potential; participates in mechanisms of resistance to oxaliplatin; increased PLK4 expression is associated with higher disease stage. High PLK4 expression in the pretreatment specimens of the rectal cancer patients is associated with poor response to neoadjuvant chemoradiotherapy.	[29–32]
Liver cancer	*PLK4* is overexpressed in hepatocellular carcinoma; its overexpression promotes cell proliferation, migration and invasion, while downregulation suppresses these cell behaviors; high *PLK4* expression is associated with shorter overall and disease-free survival in patients [33, 34]. Other studies suggest that PLK4 mRNA and protein levels are decreased in hepatocellular carcinoma compared with noncancerous tissues and that the lower levels are associated with poorer clinicopathological and shorter survival parameters; PLK4 silencing increases cell growth *in vitro* [35, 36].	[33–36]
Pancreatic cancer	High levels of *PLK4* expression are associated with poor overall survival in pancreatic ductal adenocarcinoma patients.	[37]
Bladder cancer	PLK4 mRNA and protein are overexpressed in bladder cancer cell lines and tissues; downregulation of PLK4 inhibits cell proliferation and growth; high levels of *PLK4* expression correlate with shorter overall survival (OS).	[38]
Renal cancer	*PLK4* mRNA expression levels are significantly higher in clear cell renal cell carcinoma cells compared with paracancerous tissues; its expression is related to immune infiltration and cytokines that are associated with immune suppression; high *PLK4* expression is an unfavorable prognostic factor for patient survival.	[39]
Endometrial cancer	PLK4 protein expression in endometrial tumors from patients who underwent surgical resection is associated with poor pathological and clinical characteristics; increased stability of PLK4 through its decreased ubiquitination promotes centrosome amplification and endometrial cancer cell progression; higher PLK4 protein expression levels are associated with shorter DFS and OS.	[40, 41]
Ovarian cancer	*PLK4* mRNA expression is higher in ovarian cancer tissues compared with normal ovarian tissues; in epithelial ovarian cancer, high PLK4 expression is detected in tumors of patients with the advanced pathological stage; co-expression of LIN28 homolog A (LIN28A) and PLK4 associates with poor prognosis; higher *PLK4* mRNA expression levels positively correlate with more advanced clinical stages and negatively correlate with progression-free survival (PFS) and OS.	[42, 43]
Prostate cancer	PLK4 is overexpressed in human prostate cell lines and tumors; overexpression of *PLK4* correlates with higher Gleason scores and worse DFS.	[44]
Thyroid cancer	PLK4 mRNA and protein expression is higher in some anaplastic thyroid carcinoma cell lines compared with the normal thyroid epithelial cells; PLK4 is elevated in papillary thyroid carcinoma patients’ tumor tissues compared with normal thyroid gland tissues; high PLK4 expression is associated with poorer clinicopathologic parameters, correlates with shortened DFS and OS and independently predicts poorer DFS.	[45, 46]
Melanoma	PLK4 is overexpressed in melanoma samples and human melanoma cell lines; elevated expression of PLK4 correlates with aggressive tumor characteristics, lymph node metastases, and shortened DFS and OS in patients with cutaneous melanoma who underwent surgical resection.	[47, 48]
Nonmelanoma skin cancers	PLK4 is significantly overexpressed in cutaneous squamous cell and basal cell carcinoma cells and tissues; knockdown of PLK4 in the cutaneous squamous cell carcinoma cells results in growth inhibition *in vitro* and reduction of tumorigenesis in a mouse xenograft model.	[49]
Leukemia	*PLK4* is highly expressed in acute myeloid leukemia (AML) patients compared with that in normal human hematopoietic stem cells; levels of PLK4 mRNA and protein are higher in AML cell lines, compared with that in peripheral blood mononuclear cells; PLK4 knockdown induces apoptosis, G2/M arrest and inhibits proliferation and colony formation in AML cells.	[50, 51]
Lymphoma	PLK4 overexpression is observed in diffuse large B-cell lymphoma (DLBCL) and is associated with poor survival in patients receiving cyclophosphamide, doxorubicin (hydroxydaunorubicin), vincristine (oncovin), and prednisone (CHOP)-based treatment.	[52]
Sarcomas	PLK4 may attenuate apoptosis and play a role in the progression of osteosarcoma [53–55]. *PLK4* mRNA is overexpressed in uterine leiomyosarcoma [56].	[53–56]
Glioblastoma	PLK4 mRNA and protein are overexpressed in glioblastoma; PLK4 promotes proliferation and tumorigenesis; increased PLK4 expression is associated with poor prognosis; PLK knockdown increases radiosensitivity and sensitivity to temozolomide and bortezomib.	[57–59]
Neuroblastoma	PLK4 mRNA and protein are overexpressed in central nervous system neuroblastoma; downregulation of PLK4 promotes apoptosis, suppresses cell migration, invasion and metastases and inhibits EMT; can modulate sensitivity to cisplatin; high PLK4 expression correlates adversely with clinical features and worse OS and PFS.	[60–62]
Other tumors	*PLK4* is upregulated in pediatric medulloblastoma, rhabdoid tumors and other embryonal tumors of the brain.	[63–65]

PLK4 asserts its pro-tumorigenic function through its participation in cell proliferation, growth, apoptosis, senescence, epithelial-mesenchymal transition (EMT), migration, invasion and cell cycle regulation (Table 1). High PLK4 expression might also be implicated in the suppression of the immune tumor microenvironment [73]. PLK4 has been reported to be overexpressed in various human cancers, where increased PLK4 expression is associated with worse clinicopathologic factors and poorer patient outcomes (Table 1). Hence, it can be utilized as a biomarker in various malignancies. Furthermore, PLK4 can serve as an anticancer drug target, since its pharmacologic inhibition induces antitumor immunity [74]. Inhibition of PLK4 activity by various compounds such as centrinone, centrinone B, CFI-400945, YLZ-F5, and RP-1664 has been shown to confer antitumorigenic effects and sensitize cancer cells to various chemotherapeutic drugs, targeted therapies and radiation (Table 2). Clinical trials have been designed to test PLK4 inhibitors in patients with various malignancies (Table 3).

**Table 2.** Preclinical evidence of antitumor activity of PLK4 inhibitors in non-breast cancer malignancies.

Tumor type	Anticancer effects	References
Lung cancer	CFI-400945 treatment causes polyploidy, tumor growth inhibition and apoptosis; synergizes with CDK2 (cyclin-dependent kinase 2) inhibitor seliciclib; leads to radiosensitization in non-small cell lung cancer cell lines and enhances radiation-induced tumor growth delay in xenograft models.	[25, 75]
Colorectal cancer	CFI-400945 treatment can decrease invasion, migration and proliferative abilities.	[31]
Liver cancer	CFI-400945 inhibits the proliferation of cancer cells with highly expressed PLK4; suppresses liver cancer progression through cell cycle inhibition and induces antitumor immunity; centrinone B treatment decreases the viability of different hepatocellular carcinoma cell lines.	[33, 34, 74]
Pancreatic cancer	CFI-400945 significantly reduces cell growth and increases survival in pancreatic cancer patient-derived mouse xenograft models.	[76]
Bladder cancer	CFI-400945 treatment reduces cell proliferation and induces G1 arrest.	[38]
Ovarian cancer	PLK inhibitor YLZ-F5 inhibits ovarian cancer cell proliferation by inducing apoptosis and mitotic defects.	[43]
Prostate cancer	CFI-400945 and centrinone B inhibit cell growth, viability and colony formation and induce cell cycle arrest and senescence.	[44]
Thyroid cancer	Centrinone treatment leads to decreased cell viability, induction of apoptosis, G2/M cell cycle arrest and synergizes sorafenib’s antitumor effects in anaplastic thyroid carcinoma cells.	[45]
Melanoma	Centrinone B significantly decreased melanoma cells proliferation and induced apoptosis.	[47]
Nonmelanoma skin cancer	Centrinone and CFI-400945 treatments decrease cell viability, proliferation, and clonogenic survival in nonmelanoma skin cancer cell lines.	[49]
Leukemia	Centrinone treatment leads to a decrease in colony forming ability and induces apoptosis and G2/M arrest in acute myeloid leukemia (AML) cells [50, 51]. CFI-400945 (and centrinone B) show antitumorigenic effects in p53-mutated AML cells, such as suppression of cell growth, induction of DNA damage responses, apoptosis, senescence and polyploidy, activation of the immune response and remodeling of histone methylation [77]. CFI-400945 treatment leads to antitumor effects in leukemia cell lines and AML xenograft models [78].	[50, 51, 77, 78]
Multiple myeloma	CFI-400945 synergizes bortezomib in decreasing cell viability and enhancing apoptosis via PI3 K/AKT signaling; centrinone B shows anticancer effects and potentializes effects of lenalidomide.	[79, 80]
Lymphoma	CFI-400945 shows significant antitumor activity in various lymphoma models [52, 78, 81]. In diffuse large B-cell lymphoma (DLBCL), the combination of CFI-400945 with doxorubicin markedly delays tumor progression in DLBCL xenografts [52]. A BCL-2 (B cell lymphoma 2) inhibitor, venetoclax, in combination with CFI-400945 overcomes the drug resistance and leads to a strong synergistic antitumor effect in lymphoma models [81].	[52, 78, 81]
Sarcomas	In Ewing’s sarcoma, PLK4 inhibition by CFI-400945 or centrinone leads to cell death and G2/M arrest; CFI-400945 treatment produces polyploidy [82]. CFI-400945 treatment shows antitumor activity in uterine leiomyosarcoma with DNA repair deficits and in combination with ataxia telangiectasia mutated (ATM) inhibitor, AZD0156, synergizes antitumor effects [56].	[56, 82]
Glioblastoma	CFI-400945 treatment increases sensitivity to temozolomide in patient-derived primary glioblastoma xenografts and enhances antitumor effects of bortezomib.	[58, 59]
Neuroblastoma	RP-1664, a novel PLK4 inhibitor, shows antitumor activity in neuroblastoma xenograft models and prolongs survival in a transgenic murine neuroblastoma model.	[83]
Medulloblastoma and Rhabdoid Tumors	CFI-400945 treatment decreases cell proliferation, induces apoptosis and senescence and increases susceptibility to DNA-damaging chemotherapeutic agents, such as doxorubicin and etoposide.	[84]

**Table 3.** Clinical trials on PLK4 inhibitors in cancers.

Clinicaltrials.gov ID	Intervention/treatment	Title	Phase	Cancer type	Status	Sponsor
NCT01954316	CFI-400945	A Study of CFI-400945 Fumarate in Patients With Advanced Cancer [85]	I	Advanced cancer, including, breast cancer patients	Completed	UHN
NCT03187288	CFI-400945	Study of CFI-400945 Fumarate in Patients With Relapsed or Refractory AML or MDS [78]	I	Relapsed and/or refractory AML or MDS	Completed	UHN
NCT06232408	RP-1664	LIONS (PLK4 Inhibitor in Advanced Solid Tumors)	I	Advanced solid tumors	Active, not recruiting	Repare Therapeutics
NCT04730258	CFI-400945 with or without Azacitidine	A Study of CFI-400945 With or Without Azacitidine in Patients With AML, MDS or CMML	Ib/II	AML, MDS, CMML	Active, not recruiting	Treadwell Therapeutics, Inc
NCT03385655	CFI-400945 (other drugs tested as well) [86]	Prostate Cancer Biomarker Enrichment and Treatment Selection	II	Prostate cancer	Active, not recruiting	CCTG
NCT03624543	CFI-400945	CFI-400945 in Patients With Advanced/Metastatic Breast Cancer [87]	II	HER2 negative advanced/metastatic breast cancer	Active, not recruiting	CCTG
NCT04176848	CFI-400945 with PD-L1 inhibitor durvalumab	CFI-400945 and Durvalumab in Patients With Advanced TNBC	II	Advanced/metastatic TNBC	Active, not recruiting	CCTG

AML, acute myeloid leukemia; CCTG, Canadian Cancer Trials Group; CMML, chronic myelomonocytic leukemia; ID, identification number; MDS, myelodysplastic syndrome; PD-L1, programed cell death ligand 1; PLK4, polo-like kinase 4; TNBC, triple-negative breast cancer, UHN, University Health Network, Toronto

### 1.2. PLK4 in breast cancer pathogenesis

PLK4 plays an important role in centrosome amplification in breast cancer by regulating the essential process of centriole duplication [88, 89]. Deregulation of the centrosome and its associated proteins is one of the important players in the pathogenesis and treatment responses in breast cancer [89]. The centrosomes in breast cancer cells show characteristic structural aberrations (such as an increase in the number and volume of centrosomes and an excessive increase of pericentriolar material), caused by centrosome amplification [88]. The latter in breast cancer has been linked to deregulation of PLK4 expression by transcriptional activators or modulators, such as E2F transcription factor [90] or KLF14 (Kruppel-like factor 14) [91].

Centrosome amplification drives chromosomal instability and aneuploidy and plays an important role in breast cancer biology [92, 93]. It is found in ductal carcinoma *in situ* (DCIS), a common precursor lesion for invasive breast cancer, suggesting that centrosome amplification is an early event in breast cancer development [93]. Furthermore, PLK4 plays an important role in breast cancer progression, where it appears to provide the contextual control of cancer cell migration and motility [94] and the mediation of cytokinesis [95]. PLK4, through the induction of supernumerary centrosomes and centrosome amplification leads to an invasive breast cancer cell phenotype [92, 96]. In the most aggressive breast cancer subtype, triple-negative breast cancer (TNBC), PLK4 has been reported to promote migration and invasion of cancer cells via its modulation by FEN1 (Flap endonuclease-1 (FEN1), a nuclease important for DNA replication and repair processes [97].

PLK4 participation in breast cancer pathogenesis also involves the regulation of other cancer mechanisms such as cell death pathways. PLK4 is emerging as a protein that modulates EMT, a process by which epithelial cells acquire mesenchymal features and enhance their invasiveness and metastatic potential [98] (Table 1). In neuroblastoma, where PLK4 is found to be upregulated, its expression is negatively correlated with poor clinical and survival parameters, while PLK4 downregulation suppresses neuroblastoma cells migration and invasion by inhibiting EMT via the PI3 K/Akt signaling [61]. In colorectal cancer, high PLK4 expression promotes EMT and tumor progression via regulation of the Wnt/β-catenin pathway [30]. In PLK4-depleted HeLa cells a shift from a classical mesenchymal to a more epithelial phenotype has been observed [99]. Since, PLK4 depletion leads to a substantial decrease of breast cancer cells’ invasiveness into surrounding tissues *in vivo* [99], it is plausible that these effects are modulated by PLK4’s regulation of EMT [100]. Indeed, PLK4 overexpression induces hybrid EMT phenotype in p53 knockout non-tumorigenic mammary epithelial cells [101]. While there is lack of studies in terms of the role of PLK4 in breast cancer, one study confirms a potentially important role of PLK4 in breast cancer [101]. Namely, nontumorigenic p53 knockout (p53KO) mammary epithelial cells, PLK4 overexpression has been shown to induce a hybrid EMT phenotype and thus to potentiate resistance to cell death by a programed cell death mechanism, anoikis [101]. Furthermore, breast cancer cells exposed to the conditioned media from PLK4-induced p53KO mammary epithelial cells also show anoikis resistance in a paracrine way [101].

Inhibition of PLK4 activity or its downregulation in breast cancer leads to tumor suppressive effects, such as decreased cancer cell viability, proliferation, growth and invasion. Breast cancer cells have been reported to be dependent on PLK4 for survival [102]. Silencing PLK4 in breast cancer cells prevents centriole duplication, induces cell death and results in a significant decrease in MDA-MB-468 TNBC tumor growth in xenograft models [102]. PLK4 silencing has been shown to significantly inhibit cell proliferation and colony forming ability of TNBC cells [103, 104]. In various breast cancer models, PLK4 depletion suppresses invasion and induces an epithelial phenotype [99, 105].

Independent of its role in centrosome biogenesis, PLK4 may also impact the tumor microenvironment to promote malignancy [101]. The role and mechanisms of PLK4 in regulating the tumor immune microenvironment, a known player in cancer development and progression, in breast cancer are not well-understood. Studies in different cancers suggest that PLK4 may regulate the tumor immune microenvironment. PLK4 is highly expressed in glioma where its higher expression associates with poorer prognosis and regulation of the tumor immune microenvironment, such as reduced infiltration of macrophages [73]. Downregulation of PLK4 expression in glioblastoma cell lines or its inhibition in intracranial tumor mouse models leads to alterations in chemokines’ expression and promotes recruitment of tumor infiltrating M1 macrophages [73]. In clear cell renal cell carcinoma, *PLK4* mRNA expression levels are significantly higher compared with paracancerous tissues and are associated with poor prognosis in patients, as well as are closely related to immunosuppressive phenotypes [39]. As mentioned previously, high levels of PLK4 expression induce anoikis resistance of both p53KO mammary epithelial and breast cancer cells exposed to their secretome, and likely modulate the tumor microenvironment to promote breast cancer tumorigenesis [101]. The purported role of PLK4 in inhibition of local immune responses is being exploited in a clinical trial investigating the combination of inhibition of PLK4 by CFI-400945 in combination with an immune checkpoint inhibitor (NCT04176848) in patients with advanced TNBC (Table 3).

Overall, the evidence strongly points to PLK4 as an important etiopathogenic factor in breast cancer.

### 1.3. Prognostic significance of PLK4 in breast cancer

Based on the studies highlighted in Table 1, PLK4 appears to have significant value as a biomarker in various cancers. Its prognostic significance in different clinical scenarios in breast malignancies has been reported. Centrosome amplification is associated with reduced all-cause and breast cancer-specific overall survival (OS), reduced recurrence-free survival (RFS), high-risk subtypes (such as TNBC), and advanced grade and stage of the disease [68]. Centrosome amplification is frequently observed in the circulating tumor cells of patients with metastatic breast cancer and can potentially serve as a marker to monitor treatment response [106]. Centrosome amplification has also been reported to have prognostic significance in local recurrence and relapse-free survival in DCIS [107].

PLK4 is commonly overexpressed in breast cancer. *PLK4* mRNA in breast cancer cell lines showed significantly higher levels relative to primary human mammary epithelial cells (HMEC) [102]. Using Oncomine database analysis, *PLK4* mRNA expression has been reported to be elevated in breast cancer patients’ tumors compared with healthy tissues [108]. Expression of *PLK4* mRNA has been reported to be upregulated in ∼87% of patients with breast cancer [109]. In another study, *PLK4* overexpression was found in 26% of all breast tumors and 48% of TNBC [102]. PLK4 protein expression was significantly upregulated in cancer tissues of breast ductal carcinomas as compared with the adjacent normal tissues [91]. PLK4 was found to be overexpressed in aggressive TNBC compared with non-TNBC patient samples associating with centrosome amplification, which strongly correlates with tumor aggressiveness and metastases [110]. Several studies report that PLK4 expression positively correlates with various worse clinical outcomes and clinicopathological parameters, such as higher incidence of lymph node and distant metastases and poorer OS, progression-free survival (PFS) and RFS in breast cancer patients [37, 108, 109, 111]. In breast cancer patients with no distant metastases receiving neoadjuvant chemotherapy, a positive correlation between PLK4 expression in cancerous tissues and occurrence of surrounding recurrence and distant metastases has been reported [109] In a stratified analysis, high expression of *PLK4* mRNA has been reported to be associated with poor outcomes in patients with hormone receptor-positive, HER2-negative tumors [111]. Thus, PLK4 expression can serve as a predictor for disease recurrence, survival and response to treatment.

PLK4 genetic mutations of unknown clinical significance in breast cancer samples have also been reported [108]. Overall, this data points to the possibility that PLK4 can serve as a valuable biomarker and drug target in breast cancer.

### 1.4. Pharmacologic inhibition of PLK4 in breast cancer

Given the significant role of PLK4 in cancer pathogenesis, a substantial effort has been made to develop compounds targeting this molecule [6], and these are described below.

#### 1.4.1. CFI-400495

The initial insights into the development of small-molecule inhibitors against PLK4 were made almost two decades ago [112]. Subsequent work led to the discovery of a first-in-class orally available PLK4 kinase inhibitor CFI-400945, which has a potent anticancer effect in breast, lung and colon cancer models and possesses a satisfactory pharmacological profile [113]. However, this compound has off-target effects on other kinases such as Aurora Kinase B (AURKB, 8.5-fold selective for PLK4 over AURKB), potentially contributing to its anticancer effects at higher doses. CFI-400945 has also been shown to have a bimodal effect on centriole numbers, increasing centriole numbers at lower doses and suppressing centrosome duplication at higher doses (Fig. 1c) [102]. It is proposed that at lower concentrations of CFI-400945 PLK4 is not sufficiently autophosphorylated for degradation and the increased levels of PLK4 lead to centriole overduplication [102]. When PLK4 activity is fully inhibited at higher drug concentrations, centriole duplication is blocked irrespective of PLK4 levels [102]. At higher doses, of CFI-400945 might also lead to cytokinesis failure and ensuing polyploidy, which could be related to off-target effects of the drug on AURKB inhibition [114]. Regardless, at high or low concentrations, CFI-400945 causes mitotic abnormalities, aneuploidy and genomic instability.

Preclinical therapeutic assessment of CFI-400945 in various cancer models, including breast cancer, showed that this is a potent inhibitor with effects consistent with PLK4 kinase inhibition (e.g. dysregulated centriole duplication and mitotic defects), cell death and tumor growth inhibition in mouse xenograft models [102]. Subsequently, its anticancer efficacy was shown in various cancer types (Table 2). CFI-400945 has shown significant anticancer effects in TNBC cell lines and patient-derived organoids [103, 115]. In mouse MDA-MB-231 xenograft models, CFI-400945 decreased tumor growth and significantly extended mouse tumor humane endpoint survival without substantial side effects, compared with controls [115]. Based on strong preclinical data, CFI-400495 has entered the clinical investigation stage in breast cancer (Table 3).

CFI-400495 has been tested in a Phase I dose-escalation trial (NCT01954316) in patients with advanced solid tumors, including breast cancer [85]. The drug was well tolerated with dose-dependent neutropenia being the most common high-grade adverse event [85]. Phase II studies have also been initiated in patients with advanced/metastatic breast cancer with CFI-400945 monotherapy (NCT03624543) or in combination with the immunotherapeutic agent durvalumab (NCT04176848) (Table 3). While final analysis of the latter trial is ongoing, it has been reported that CFI-400945 and durvalumab were well tolerated in the heavily pretreated and PD-L1 unselected advanced TNBC population; however, no responses were observed and the pre-specified threshold for anti-tumor activity was not met, resulting in the closure of the trial for accrual [116]. An open label, multicentre, Phase II study in metastatic TNBC, ER+/HER2- PTEN (phosphatase and tensin homolog) low or ER+/HER2- PTEN intact breast cancer patients treated with CFI-400945 was initiated, showing that the drug was well tolerated with a moderate incidence of uncomplicated neutropenia [87] (Table 3). The TNBC cohort of this trial showed lack of response, and this arm was closed to further accrual. Responses in the ER+/HER2- patient cohort were encouraging and are awaiting final analysis of the results [87]. Early-stage clinical trials of CFI-400945 in other cancers have also been initiated and conducted, showing promising results in Phase I studies for patients with relapsed/refractory acute myeloid leukemia and higher-risk myelodysplastic neoplasms (NCT03187288) [78]. (Table 3).

#### 1.4.2. Centrinone and centrinone B

Centrinone and centrinone-B have been developed as highly selective PLK4 inhibitors that exhibited high selectivity against many kinases (e.g. > 1000-fold selectivity for PLK4 over Aurora A/B) [117]. Centrinones cause centrosome depletion and growth arrest of normal human cells in a senescence-like G1 state in a p53-dependent manner. However, cancer cells, continue to proliferate despite centrinone-induced centrosome depletion, albeit with substantially reduced mitotic fidelity [117]. Centrinones showed potent anticancer effects in various cancer models (Table 2). In MDA-MB-231 TNBC cells, centrinone B inhibits cell proliferation [103] and suppresses wound healing and directional migration of cancer cells [99].

Sensitivity of cancer cells to PLK4 inhibition appears to be dependent on the levels of TRIM37 (Tripartite motif-containing protein 37) ubiquitin ligase [118, 119]. TRIM37 plays an important function in centrosome regulation and is commonly amplified in breast cancer [120].When PLK4 is inhibited by centrinone, cell division takes place without centrosome duplication, leading to delayed, acentrosomal spindle assembly [117]. High TRIM37 levels result in inhibition of acentrosomal spindle assembly leading to mitotic failure and inhibition of cell proliferation, thus augmenting the effects of centrinone inhibition [118]. In MCF-7 cells with amplified TRIM37, CFI-400945, centrinone or an AURKB inhibitor potently decreased clonogenic survival [119]. In this model, depletion of TRIM37 only restored the proliferation of centrinone-treated cells. Since centrinone is a highly selective PLK4 inhibitor (as compared with CFI-400945), this suggests that inhibitor selectivity toward PLK4 is essential for the synthetic lethal killing of cells overexpressing TRIM37 [119]. However, metabolic instability and the lack of oral bioavailability of centrinone B appears to be a limiting factor for further clinical development [121, 122]. Other new compounds targeting PLK4 have been shown to specifically lead to anticancer effects in TRIM37-amplified breast cancer models [123, 124].

#### 1.4.3. RP-1664

Using centrinone B as a starting point, another PLK4 inhibitor, RP-1664, has been developed that demonstrates potent and selective PLK4 inhibition with good oral bioavailability [125]. RP-1664 disrupts centriole biogenesis in cancer cells and shows excellent efficacy in pre-clinical TRIM37-amplified models, including breast cancer [83, 125]. RP­-1664 has entered a Phase I clinical trial stage in patients with selected advanced solid tumors based on promising preclinical data (NCT06232408) (Table 2). This compound is reported to be especially effective in TRIM37 high tumors [126]. The latter are dependent on centrioles for successful cell division, allowing PLK4 inhibition through disregulation of centrioles to lead to anticancer effects.

#### 1.4.4. YLT-11

YLT-11 is another oral PLK4 inhibitor, which inhibits growth of human breast cancer cell lines and induces apoptosis by causing dysregulated centriole duplication and mitotic defects [104]. In MCF-7, MDA-MB-468, and MDA-MB-231 mouse xenograft models, oral administration of YLT-11 significantly inhibited tumor growth in a dose-dependent manner and was well tolerated in experimental animals [104]. Other similar compounds have been described that have good anticancer potency in breast cancer cell lines and xenograft models [6, 127].

#### 1.4.5. Other compounds

In addition, several other compounds with PLK4 inhibitory activity have been recently reported to show potent anticancer effects in breast cancer [123, 124, 128–132], ovarian cancer [43] and leukemia models [133]. Finally, a proteolysis targeting chimera (PROTAC) against PLK4 has been developed [124]. PROTACs are designed to bind the target protein and through the binding platform for E3 ubiquitin ligase, recruit the latter for degradation of the target [6]. This PLK4 PROTAC degrader showed high potency and selectivity, and PLK4 degradation in TRIM37-amplified MCF-7 cells and xenograft models [124]. For a detailed review of the developments of new anti-PLK4 compounds, please refer to Lei et al., 2024 [6]. Further pre-clinical and clinical development of these compounds might provide additional novel drugs against PLK4 to be tested in breast cancer.

### 1.5. PLK4 as a target to assess and modify treatment response in breast cancer

In addition to being a treatment target, PLK4 may also be valuable as a biomarker of response to cancer treatment including in breast cancer [134] and rectal cancer [29] (Table 1). For example, PLK4-driven centrosome amplified breast tumor cells have been reported to be highly sensitive to inhibitors of the STAT3 (signal transducer and activator of transcription 3) protein [135], an emerging potential therapeutic target in breast cancer [136]. PLK4 expression is also reported to be a negative predictor of response to taxane-based neoadjuvant chemotherapy in breast cancer [109].

PLK4 also holds promise as a target for augmenting the therapeutic effects of other treatment modalities, such as chemotherapy, targeted therapy, immunotherapy and radiotherapy (Table 2). PLK4 upregulation decreases sensitivity to chemotherapeutic agents, while its downregulation leads to opposite effects. PLK4 has been reported to modulate sensitivity to various chemotherapeutic agents such as temozolomide in glioblastoma [58]; cisplatin in neuroblastoma [62] and oxaliplatin in colorectal cancer [32] (Table 2). PLK4 inhibition by CFI-400945 has been shown to have a synergistic anticancer effect with temozolomide in glioblastoma patient-derived xenograft mouse models [58]; etoposide or doxorubicin in medulloblastoma and rhabdoid tumors [84]; proteasome inhibitor bortezomib in multiple myeloma [79] and glioblastoma [59]; ATM inhibitor AZD0156 in uterine leiomyosarcoma [56]; and CDK2 (cyclin-dependent kinase 2) inhibitor seliciclib in lung cancer [25]. Inhibition of PLK4 by centrinone also exhibits synergistic anticancer effects with sorafenib in anaplastic thyroid carcinoma [45]. Inhibition of PLK4 by CFI-400945 leads to overcoming oxaliplatin resistance in colorectal cancer mouse xenograft models [32]. Inhibition of PLK4 by centrinone B also helps to overcome resistance to lenalidomide in multiple myeloma cells [80]. Some lymphoma cell lines show sensitivity to CFI-400945 while others are resistant to its antitumorigenic effects [81]. A BCL-2 (B cell lymphoma 2) inhibitor venetoclax, in combination with CFI-400945 overcomes drug resistance and leads to a strong synergistic antitumor effect in lymphoma models [81] (Table 2). A potential synergistic effect between immune checkpoint inhibitors and PLK4 inhibition, which through DNA replication errors, can generate neoantigens, might also be promising in cancer treatment [6]. This strategy of PLK4 inhibition in combination with an immune checkpoint inhibitor is being explored in a Phase II clinical trial of CFI-400945 in combination with durvalumab in patients with advanced/metastatic TNBC (NCT04176848) (Table 3).

Numerical chromosomal instability, such as that caused by PLK4 deregulation, might mediate responses of cancer cells to radiation treatment [137]. PLK4 has been reported to be associated with radioresistance in malignancies [57]. Knockdown of PLK4 leads to the radiosensitivity of various cancers, such as glioblastoma [57] (Table 1). Inhibition of PLK4 by CFI-400945 enhances radiosensitivity in lung cancer [75] (Table 2). Radiation treatment might impact the expression of the PLK4 gene, since PLK4 mRNA was found to be downregulated at 72 hours after 2 or 4 Gy radiation treatment in MCF-7 cell lines but not in MDA-MB-231 cells [111]. PLK4 inhibition by CFI-400945 in breast cancer *in vivo* and *in vitro* TNBC models has been shown to augment and synergize the effects of radiation treatment [103, 115], through mechanisms that potentially involve overamplification of centrioles [103] and alterations in DNA damage response and cell cycle regulation [138]. Combination treatment of CFI-400945 with radiation leads to significantly improved mouse tumor humane endpoint survival and reduced tumor growth compared with control or single-agent treatments [115]. CFI-400945 might cause off-target effects of inhibition by AURKB. AURKB inhibition by its inhibitor AZD1152 also enhanced the effects of radiotherapy in TNBC [139], suggesting that the radiosensitizing effect of CFI-400945 might be at least in part conferred by inhibition of this protein. However, highly specific centrinone B or PLK4 silencing has also been shown to provide combinatorial anticancer effects when combined with radiation in TNBC cell lines [103]. Together with studies from other cancer types, these results strongly suggest that PLK4 is an important new target for enhancing the sensitivity of breast cancer cells to radiotherapy.

## 2. Conclusions and future directions

Overall, this review highlights that PLK4 is a promising therapeutic target for breast cancer and precision oncology. It has great utility to be used as a biomarker in prognostication and assessment of treatment response in invasive and *in situ* breast carcinomas. PLK4 protein or RNA expression can be further explored as a biomarker for assessing patient prognosis and responses to treatment, through prospective clinical studies in patients with non-metastatic and metastatic breast cancer. Given that PLK4 expression or inhibition correlates with responses to chemotherapy and radiotherapy, further studies of PLK4 expression as a biomarker for treatment response might allow for better stratification of patient treatments, especially in metastatic patient populations. Since the most need for biomarker development and worse clinical outcomes exist in TNBC subtype, more focused clinical studies in this patient cohort are of elevated urgency.

Moreover, PLK4 appears to be a promising therapeutic target. The arsenal of its inhibitors is increasing. Existing PLK4 inhibitors show great promise in the treatment of breast cancer, with their oral availability constituting one of the major advantages. Continuous PLK4-tageting drug development shows promise in terms of expanding the arsenal of PLK4 inhibition and various clinical trials with PLK4 inhibitors have been initiated. The best studied PLK4 inhibitor, CFI-400945 shows promise as a novel treatment for metastatic breast cancer patients. Despite off-target effects of CFI-400945 it still holds promise to be evaluated in a clinical setting, and might be particularly efficacious through inhibition of both PLK4 and AURKB [114]. Inhibition of the latter appears to be a promising strategy for breast cancer treatment by itself. [139, 140]. Thus, CFI-400945 may hold an advantage of providing a double-hit in cancer treatment, through inhibition of both proteins.

Synergistic anticancer effects of CFI-400945 with radiotherapy observed in preclinical models of lung [75] (Table 2) and breast cancers [103, 115, 138] provide an opportunity to develop new multimodality treatment approaches with PLK4 inhibition and radiotherapy to augment the anticancer efficacy of the latter, while providing potential advantages of the systemic treatment through the PLK4 inhibitor. This approach would be particularly advantageous in heavily pretreated metastatic breast cancer patients who are planned to undergo radiotherapy for local control of the metastatic sites and warrants further investigation in clinical trials. Furthermore, combination treatments of PLK4 inhibitors with other therapeutic agents in breast and other cancers might be of value, especially to augment tumor responses to immunotherapy in patients with metastatic breast cancer, an approach that is already being implemented in clinical trials (Table 3). PLK4 inhibition might be a useful therapeutic strategy, especially for TRIM37 amplified breast tumors, as a stand-alone modality or in combination with other treatment modalities to synergize their anticancer effects and/or overcome resistance. A novel compounds, RP-1664, might prove to be efficacious in these patient populations, and showed a great promise in preclinical studies with results of clinical investigations of RP-1664 being highly awaited.

In conclusion, further investigations of the role of PLK4 in breast cancer pathogenesis and its response to treatment are warranted to better understand breast cancer biology and to develop better treatment strategies and novel therapeutic approaches in breast cancer. Further translational and clinical studies of PLK4 inhibitors would provide a foundation for the development of novel biomarkers and effective treatment strategies in this, one of the most morbid and lethal cancer types in women worldwide.

## Data Availability

No new data were generated or analyzed in support of this research.
